# Prevalence and adverse perinatal outcomes of anaemia in the third trimester of pregnancy in Hebei Province, China

**DOI:** 10.1093/inthealth/ihad028

**Published:** 2023-04-24

**Authors:** Mei-Ling Tian, Guo-Juan Ma, Li-Yan Du, Yuan-Ge Xiao, Ying-kui Zhang, Zeng-Jun Tang

**Affiliations:** Depart ment of Obstetrics and Gynecology, Hebei General Hospital, Shijiazhuang, China; Depart ment of Obstetrics and Gynecology, Hebei General Hospital, Shijiazhuang, China; Department of Information Management, Hebei Center for Women and Children's Health, Shijiazhuang, China; Depart ment of Obstetrics and Gynecology, Hebei General Hospital, Shijiazhuang, China; Department of Information Management, Hebei Center for Women and Children's Health, Shijiazhuang, China; Depart ment of Obstetrics and Gynecology, Hebei General Hospital, Shijiazhuang, China

**Keywords:** adverse perinatal outcomes, anaemia, China, pregnant, prevalence

## Abstract

**Background:**

Anaemia in pregnancy is one of the most frequent complications related to pregnancy and is a public health concern. This article examines the prevalence of anaemia in the third trimester of pregnancy and the associations between anaemia and adverse perinatal outcomes in Hebei Province, China.

**Methods:**

We used SPSS software to describe the incidence of anaemia in the third trimester of pregnancy in Hebei Province and analysed the clinical characteristics in anaemic patients and the relationship between anaemia and adverse pregnancy outcomes.

**Results:**

The overall prevalence of anaemia in the third trimester of pregnancy was 35.0% in Hebei Province. The prevalence of anaemia in the population with a high education level was lower than that in the population with a low education level. The incidence rate in rural areas was higher than that in urban areas. After adjustment for confounding factors, anaemia in the third trimester of pregnancy is an independent risk factor in terms of placenta previa, placental abruption, uterine atony, pre-eclampsia, gestational diabetes mellitus, heart disease, postpartum haemorrhage, premature birth, laceration of birth canal, puerperal infection, caesarean section and large for gestational age.

**Conclusions:**

The prevalence of anaemia in the third trimester of pregnancy is associated with an increased risk of adverse perinatal outcomes. A comprehensive approach to prevent anaemia is needed to improve maternal and child health outcomes.

## Introduction

Anaemia affects approximately one-third of the world's population; half the cases are due to iron deficiency.^1,2^ The global prevalence of anaemia in non-pregnant and pregnant women is 29% and 38%, respectively.^3^ Anaemia in pregnancy is one of the most frequent complications related to pregnancy.^4^ Anaemia in pregnancy has become a public health problem affecting both developing and developed countries. According to the WHO guidelines, anaemia in pregnancy is defined as a condition in which haemoglobin level is <110 g/L. Anaemia is generally considered to be a risk factor for adverse pregnancy outcomes, which may lead to complications and threaten the lives of both mother and infant, such as postpartum hemorrhage (PPH), heart failure, preterm birth,^5–8^ low birth weight,^9^ foetal impairment and neonatal mortality.^10,11^ In this study we assessed the prevalence of anaemia and evaluated the maternal and foetal outcomes among anaemic and non-anaemic women in Hebei Province, China.

## Methods

### Data sources

From 1 January 2014 to 31 December 2021, 394 898 deliveries from the monitoring information management system for pregnant women in 22 hospitals of Hebei Province China were collected for the study. The inclusion criteria covered delivery >28 wk, delivery age (14–55 y) and singleton live birth. The exclusion criteria included stillbirth, multiple births and incomplete data. A total of 362 023 deliveries were included; 126 636 pregnant women were diagnosed with anaemia. All methods were performed in accordance with the relevant regulations. We secured informed consent from all subjects.

### Definitions

Based on WHO criteria, we defined anaemia in pregnancy as Hb<110 g/L.^12^

### Statistical analysis

The statistical analyses were performed using SPSS 21.0 (Chicago, IL, USA). The data described are presented as mean±SD for continuous variables. The counting data are expressed as percentages. The T-test was used for the comparison between groups of measurement data that conform to normal distribution, the F-test is used for the comparison between groups of counting data that do not conform to normal distribution and the χ^2^ test is used for the comparison between groups of counting data. Bivariate and multivariate logistic regression analyses were performed to adjust for potential confounding factors. p was set at <0.05 for statistical significance.

## Results

### Prevalence of anaemia among pregnant women

**Figure 1.**
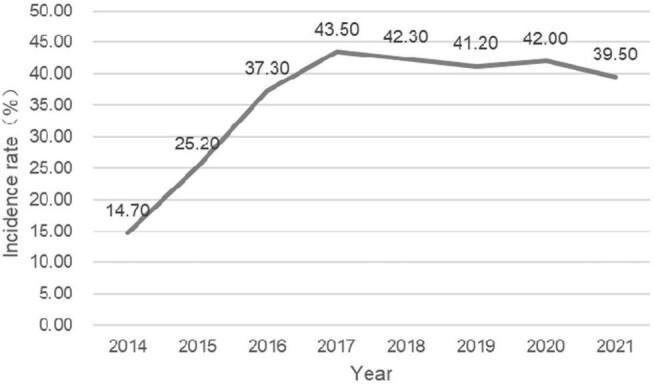
Prevalence of anaemia during pregnancy in Hebei Province from 2014 to 2021.

**Figure 2.**
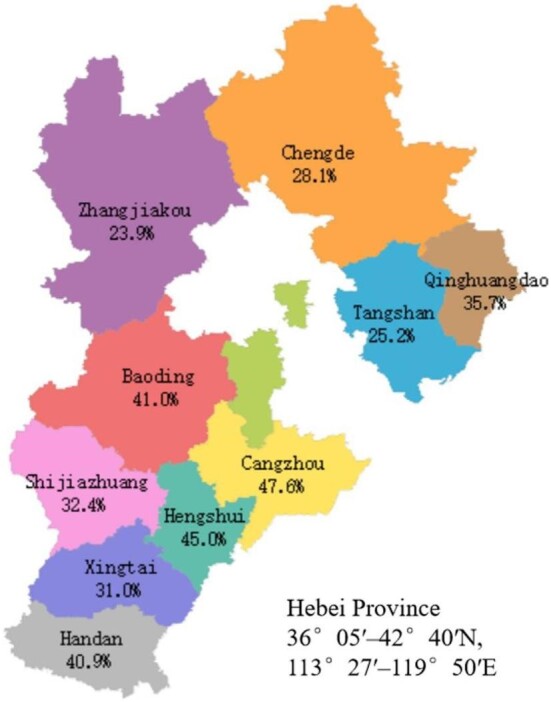
Prevalence of anaemia during pregnancy in Hebei Province in different areas from 2014 to 2021.

**Figure 3.**
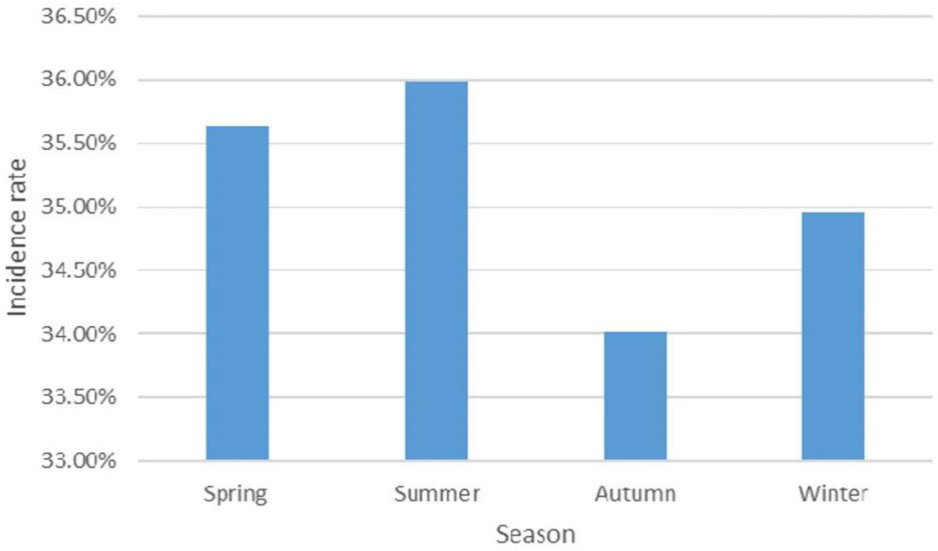
Prevalence of anaemia during pregnancy in Hebei Province in different seasons from 2014 to 2021.

Data from 362 023 pregnant women were used for analysis. The number of participants in the anaemia groups was 126 636. The prevalence of anaemia was 35.0% (126 636/362 023). The demographic and obstetric difference between the two groups was statistically significant in terms of maternal age, education level, parity, the grade of delivery hospital and a history of caesarean section (p<0.05). The incidence of anaemia in pregnant women aged 20–34 y was significantly lower than that in pregnant women aged 15–19 y and ≥35 y. The prevalence of anaemia in the population with a high education level was lower than that in the population with a low education level. The incidence rate in rural areas was higher than that in urban areas. There was no significant difference regarding marital status (p>0.05) (Table 1). The incidence of anaemia during pregnancy showed an upward trend from 2014 to 2017, and a downward trend from 2017 to 2021 (Figure 1). Sorted by city, the top three cities with anaemia during pregnancy were Cangzhou (47.6%), Hengshui (45.0%) and Baoding (41.0%) (Figure 2). The lowest incidence of anaemia during pregnancy is in autumn and the highest is in summer (Figure 3).

**Table 1.** The maternal demographic characteristics of participants in Hebei Province from 2014 to 2021 (N [%])

Characteristic	Anaemic group	Non-anaemic group	F/χ2	p
**Maternal age (y)**	29.43±4.57	29.26±4.55	8.527	0.004
**Maternal age (y)**			24.75	<0.001
15–19	1024(37.10)	1739(62.90)		
20–34	108 189(34.80)	202 463(65.20)		
35–39	14 691(35.90)	26 235(64.10)		
≥40	2732(35.600)	4950(64.40)		
**Educational status**			1148.41	<0.001
College and above	44 599(31.60)	96 381(68.40)		
Senior	40 121(36.80)	69 047(63.20)		
Junior and below	41 916(37.50)	69 959(62.50)		
**Marital status**			0.15	0.700
Married	126 126(35.00)	234 459(65.00)		
Unmarried	510(35.50)	928(64.50)		
**Parity**			1445.45	<0.001
0	50 873(31.60)	110 049(68.40)		
≥1	75 718(37.70)	125 238(62.30)		
**Region**			2279.78	<0.001
urban	54 751(31.10)	121 346(68.90)		
rural	71 885(38.70)	114 041(61.30)		
**History of caesarean section**			3383.47	<0.001
No	87 176(32.30)	182 769(67.70)		
Yes	39 329(42.90)	52 351(57.10)		

### The risk of adverse pregnancy outcomes because of anaemia

For maternal outcomes, we found that the prevalence of placenta previa, placental abruption, pre-eclampsia, gestational diabetes mellitus (GDM), premature delivery, laceration of birth canal, caesarean section and heart disease were statistically different between the two groups (p<0.05). There was no statistical difference in puerperal infection (p>0.05). For infant outcomes, there were significant differences in terms of birth weight (p<0.05) (Table 2).

**Table 2.** Adverse pregnancy outcomes in anaemic and non-anaemic women in Hebei from 2014 to 2021 (N [%])

Variables	Anaemic group	Non-anaemic group	χ2	p
**Maternal**				
**Placenta previa**			67.38	<0.001
No	125 964(34.90)	234 569(65.10)		
Yes	672(45.10)	818(54.90)		
**Placental abruption**			50.98	<0.001
No	126 209(35.00)	234 889(65.00)		
Yes	427(46.20)	498(53.80)		
**Uterine atony**			2781.65	<0.001
No	123 509(34.50)	234 245(65.50%		
Yes	3127(73.20)	1142(26.80)		
**Pre-eclampsia**			16.61	<0.001
No	123 099(34.90)	229 349(65.10)		
Yes	3537(36.90)	6038(63.10)		
**GDM**			93.68	<0.001
No	117 021(34.80)	219 545(65.20)		
Yes	9615(37.80)	15 842(62.20)		
**Postpartum haemorrhage**			2722.299	<0.001
No	123 687(34.50)	234 364(65.50)		
Yes	2949(74.20)	1023(25.80)		
**Premature delivery**			30.52	<0.001
No	120 040(35.10)	222 094(64.90)		
Yes	6596(33.20)	13 293(66.80)		
**Laceration of birth canal**			91.65	<0.001
No	126 385(35.00)	235 195(65.00)		
Yes	251(56.70)	192(43.30)		
**Puerperal infection**			0.13	0.719
No	126 609(35.00)	235 341(65.00)		
Yes	27 (37.00)	46 (63.00)		
**Caesarean section**			2716.42	<0.001
No	53 147 (30.70)	120 143 (69.30)		
Yes	73 488 (38.90)	115 237 (61.10)		
**Heart disease**			10.18	0.001
No	126 358(35.00)	234 738(65.00)		
Yes	278(30.00)	649(70.00)		
**Newborn weight**			713.03	<0.001
Normal	89 354 (34.90)	166 900 (65.10)		
SGA	18 066 (31.60)	39 024 (68.40)		
LGA	19 216 (39.50)	29 463 (60.50)		

Abbreviations: GDM, gestational diabetes mellitus; LGA, large for gestational age; SGA, small for gestational age.

**Figure 4.**
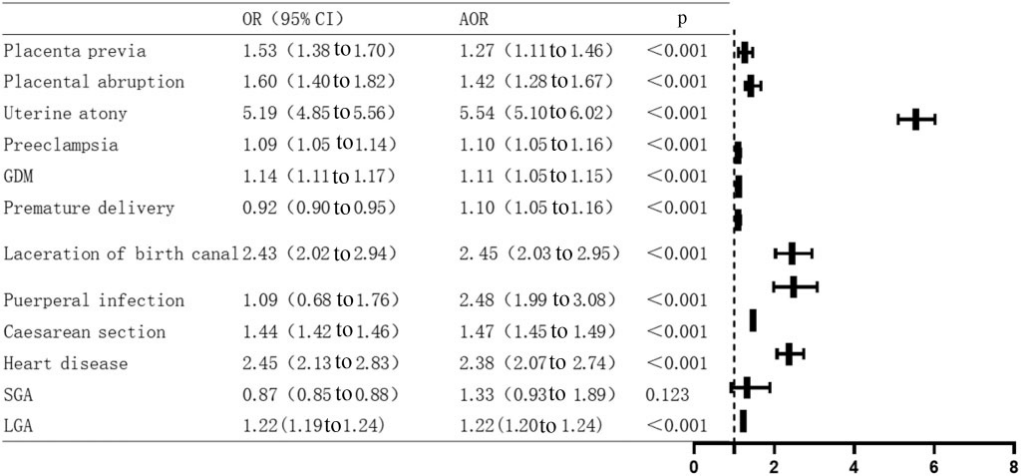
Univariate and multivariate logistic regression model of maternal and infant outcomes of anaemic individuals. AOR, adjusted OR.

Univariate logistic regression analysis showed that anaemia in the third trimester of pregnancy was a risk factor for placenta previa, placental abruption, uterine atony, pre-eclampsia, GDM, postpartum haemorrhage, premature birth, laceration of birth canal, caesarean section, large for gestational age (LGA) and heart disease (p<0.05). After adjustment for age, gravidity, parity and education level, the multivariate logistic regression analysis model showed that the incidence of placenta previa in anaemic women was 1.27 times higher than that in non-anaemic women (OR=1.27, 95% CI 1.11 to 1.46). Anaemic pregnant women were also at significantly increased risk of most adverse pregnancy outcomes in terms of placental abruption (OR=1.42, 95% CI 1.28 to 1.67), uterine atony (OR=5.54, 95% CI 5.10 to 6.02), pre-eclampsia (OR=1.10, 95% CI 1.05 to 1.16), GDM (OR=1.11, 95% CI 1.05 to 1.15), postpartum haemorrhage (OR=5.81, 95% CI 5.40 to 6.24), premature birth (OR=1.10, 95% CI 1.05 to 1.16), laceration of birth canal (OR=2.45, 95% CI 2.03 to 2.95), puerperal infection (OR=2.48, 95% CI 1.99 to 3.08), caesarean section (OR=1.47, 95% CI 1.45 to 1.49), LGA (OR=1.22, 95% CI 1.20 to 1.24) and heart disease (OR=2.38, 95% CI 2.07 to 2.74) (Figure 4).

## Discussion

Anaemia in pregnancy is one of the most common complications during pregnancy, and increases the occurrence of adverse pregnancy outcomes. It has become a global public health problem, not only in developing, but also in developed countries.^13^ The overall prevalence of anaemia in China was 23.5%.^14^ In northern China, the majority of anaemia is caused by iron deficiency.^15^ In this study, the overall prevalence of anaemia in Hebei Province was 35.0%, which was higher than the average incidence in China. It remains a severe health problem in Hebei Province. On one hand, it may be related to eating habits. Alternatively, with the promotion of perinatal healthcare knowledge, obstetric medical workers are increasingly familiar with and attach importance to screening anaemia. Therefore, we should improve our eating habits and provide early screening for anaemia and early supplements of iron after diagnosis.

The incidence of anaemia reached a turning point in 2017 in Hebei Province. The incidence of anaemia during pregnancy showed an upward trend from 2014 to 2017, and a downward trend from 2017 to 2021. This may be due to the fact that ever since the introduction of the two-child policy in 2016, the state has paid more attention to perinatal nutrition and screening anaemia during pregnancy, along with early supplementation of iron. Alternatively, the proportion of multipara increased after the change of fertility policy. The experience of previous pregnancy made pregnant women pay more attention to nutrition knowledge and iron supplements during pregnancy.

Previous studies have shown an association of anaemia with a low education status.^13^ We also found that the prevalence of anaemia in the population with a high education level was lower than that in the population with a low education level. This may be due to the high level of knowledge, higher acceptance and attention to healthcare, as well as the greater emphasis on nutrition and healthcare, during pregnancy. The typical childbearing age in Hebei Province is 20–34 y. Moreover, the incidence of anaemia in pregnant women at the typical age (20–34 y) was significantly lower than that in older and younger pregnant women, thus we should encourage childbearing at the typical age.

This study shows that the incidence rate in rural areas is higher than that in urban areas. This result is consistent with previous research results.^14,16^ This may be related to a lack of adequate nutrition and economic factors during pregnancy. Moreover, we demonstrated that the incidence of anaemia in Hebei Province shows obvious regional differences. The prevalence of anaemia in Cangzhou, Hengshui and Baoding is high, while that in Zhangjiakou is low. Overall, the incidence rate of anaemia is characterised by regional differences. The incidence rate of anaemia is highest in central cities, and the incidence rate of anaemia is low in northern cities. The overall trend is consistent with the level of economic distribution in Hebei Province. The reasons are analysed as follows: (1) dietary habits lead to a difference in iron intake; (2) areas with a high economic level attach importance to good nutrition during pregnancy and pay attention to the intake of food containing iron agents.

Previous studies have found that anaemia in pregnancy is associated with adverse pregnancy outcomes, such as preterm birth, hypertensive disorders and low birth weight.^17–20^ Analysis of cohort studies showed a significantly higher risk of low birth weight and preterm birth with anaemia in the first or second trimester.^17^ Women taking iron supplements less frequently had low birthweight newborns and preterm babies.^18^ A meta-analysis shows that maternal anaemia during the first trimester was associated with increased preterm birth, but not with low birth weight and hypertensive disorders of pregnancy.^19^ One study showed that iron-containing multiple micronutrient supplementation might reduce the risk of pregnancy-induced hypertension compared with only taking folic acid supplements.^20^ We found an increase of premature delivery and pre-eclampsia in anaemic women. These results were in accordance with previous findings. However, we did not find an association between anaemia and small for gestational age (SGA) in our study, while anaemia was a risk factor for LGA. This might be due to differences in research methods and objectives. In addition, pregnant women diagnosed with anaemia during the third trimester of pregnancy may consume too much food to correct anaemia, thus increasing the incidence of larger than gestational age infants. We also found an increase in uterine atony, caesarean section, postpartum haemorrhage and laceration of birth canal in anaemic women.^21,22^ This may be because anemic pregnant women are prone to uterine atony, and then the labour process is prolonged. In addition, the incidence of LGA increases, so birth canal injury, postpartum haemorrhage, puerperal infection and the caesarean section rate increase. Moreover, the immune function of pregnant women with anaemia is low and the risk of puerperal infection is increased.

In this study we found that anaemia was a risk factor for placenta previa and placental abruption. Previous studies^23–25^ reported an association between placenta previa and placental abruption with maternal iron deficiency anaemia. Both placenta previa and placental abruption are increased in women with iron deficiency anaemia. Both placenta previa and placental abruption are placental related diseases. Anaemia may cause a poor endometrial environment and affect the circulation of the placenta, which may lead to a lower placental implantation position, inadequate placental blood flow perfusion and placental ischaemia and hypoxia, thus increasing the risk of placenta previa and placental abruption.^23^ Therefore, we should pay attention to the early diagnosis and treatment of anaemia and closely monitor the situation of mother and child.

There is no clear relationship between diabetes and anaemia. The conclusions of previous studies on the relationship between diabetes and anaemia are inconsistent. Previous studies^14,26^ found that iron deficiency anaemia reduced the prevalence of GDM. This is consistent with our research.

This study studied the epidemiological characteristics of anaemia in the third trimester of pregnancy and its relationship with adverse pregnancy outcomes. However, there are no records for iron supplements and anaemia data concerning pregnant women in the first and second trimesters in this study, which may have impacted the results of adverse pregnancy outcomes for anaemia. But this study has a large amount of data and the experimental results are highly reliability.

This study showed that anaemia in pregnancy continues to be a health problem in Hebei. Therefore, we should encourage women to become pregnant at the typical age, promote early prenatal examination for pregnant women with high risk factors, publicise the importance of nutrition knowledge during pregnancy and pay attention to iron supplements during pregnancy. All these measures can reduce the occurrence of anaemia during pregnancy and ensure the safety of mothers and infants.

## Data Availability

The datasets used in the current study are available from the corresponding author on reasonable request.
